# The Effects of Auxin Transport Inhibition on the Formation of Various Leaf and Vein Patterns

**DOI:** 10.3390/plants13182566

**Published:** 2024-09-12

**Authors:** Carol L. Wenzel, David M. Holloway, Jim Mattsson

**Affiliations:** 1 Biotechnology Department, British Columbia Institute of Technology, 3700 Willingdon Avenue, Burnaby, BC V5G 3H2, Canada; 2 Mathematics Department, British Columbia Institute of Technology, 3700 Willingdon Avenue, Burnaby, BC V5G 3H2, Canada; david_holloway@bcit.ca; 3 Biology Department, Simon Fraser University, 8888 University Drive, Burnaby, BC V5A 1G3, Canada; jim_mattsson@sfu.ca

**Keywords:** convergent points, leaf complexity, polar auxin transport (PAT), venation patterning

## Abstract

Polar auxin transport (PAT) is a known component controlling leaf complexity and venation patterns in some model plant species. Evidence indicates that PAT generates auxin converge points (CPs) that in turn lead to local leaf formation and internally into major vein formation. However, the role of PAT in more diverse leaf arrangements and vein patterns is largely unknown. We used the pharmacological inhibition of PAT in developing pinnate tomato, trifoliate clover, palmate lupin, and bipinnate carrot leaves and observed dosage-dependent reduction to simple leaves in these eudicots. Leaf venation patterns changed from craspedodromous (clover, carrot), semi-craspedodromous (tomato), and brochidodromous (lupin) to more parallel patterning with PAT inhibition. The visualization of auxin responses in transgenic tomato plants showed that discrete and separate CPs in control plants were replaced by diffuse convergence areas near the margin. These effects indicate that PAT plays a universal role in the formation of different leaf and vein patterns in eudicot species via a mechanism that depends on the generation as well as the separation of auxin CPs. Computer simulations indicate that variations in PAT can alter the number of CPs, corresponding leaf lobe formation, and the position of major leaf veins along the leaf margin in support of experimental results.

## 1. Introduction

Plants have a spectacular diversity in leaf shape and complexity, both of which can vary between plant species and even within individual plants (e.g., [1,2]). Much of the diversity in leaf shape is dependent upon the extent of the outgrowth of the margin, in particular the size of the marginal blastozone and the duration of its morphogenetic potential [3,4,5,6,7,8] as well as the extent and overall distribution of growth in the leaf lamina (e.g., [9,10]).

Growth axes in leaves frequently co-align with the primary and secondary veins, suggesting a link between growth and venation and that developing procambial cells may provide a driving force behind localized leaf expansion [11,12]. Computer simulations in which growth is catalyzed at vein initiation sites in the margin indeed produce a wide range of observed leaf shapes [13]. However, there are many unanswered questions regarding how vein patterning and growth may be coordinated, particularly at the molecular level.

Molecular mechanisms are likely to include the phytohormone auxin (indole acetic acid, IAA), which is involved in both the vein patterning and growth aspects of leaf development. Primary/secondary veins and localized outgrowths of leaf margins are separated by many-cell distances of ~20–50 µm to mms (depending on stage). Understanding auxin’s role in catalyzing structures at such length scales requires understanding how auxin is spatially distributed and localized to future vascular or high-growth cells. Auxin has unique transport properties that influence this. In particular, feedback between auxin and the PINFORMED (PIN) efflux proteins ([14,15]; primarily PIN1 in *Arabidopsis*) creates an intercellular polar auxin transport (PAT) that contributes to long-range patterning.

In vein development, PAT is involved in the formation of discrete auxin concentration maxima (convergence points, CPs) in the leaf margin, from which provascular tracks extend into the leaf. Co-localized PIN1-auxin distributions have been characterized for the CP of the primary vein, as the leaf primordium emerges from the shoot apical meristem [16,17,18,19,20], as well as for the subsequent CPs associated with (i) secondary veins, teeth, and lobes in the margins of simple leaves [14,15,21] and (ii) the midveins of individual leaflets in compound leaves, for example, in *Cardamine hirsuta* [22] and tomato [23]. Following CP formation, PAT is also involved in inward vein extension and canalization (in which the early broad distributions of auxin and PIN1 narrow to produce the provascular track, e.g., [14,15,24]).

Pharmacological PAT inhibition (PATi)—for example, by treatment with NPA, N-(1-Naphthyl) phthalamic acid [25,26], which competes with auxin binding to PIN1 [27]—or *PIN* mutations [28] have been shown to affect both of these aspects of vein development in *Arabidopsis* simple leaves: affecting CP formation [25,26] and delaying vein extension [25,26] and canalization [14,15]. In compound leaves, PATi has been shown to affect CPs in *Cardamine* (auxin; [22]) and in tomato (PIN1; [23]).

PATi also influences leaf shape, rounding the simple leaves of *Arabidopsis* [25,26,28] and inducing more marked effects in compound leaves, for example, reducing leaf complexity in tomato [23,29,30], *Cardamine* [22], and pea [31], producing simpler leaves with fewer or no leaflets.

These results point to a shared mechanism for the auxin localization involved in vein patterning and morphogenesis in plants with simple and compound leaves, but questions remain. These include: does vein patterning in compound leaves have a similar response to PATi as in simple leaves; what is the correspondence between morphological changes and vein pattern changes; and can PATi responses in morphologically complex compound leaves provide more information on this relation than in simple leaves?

To begin to address these questions, we conducted PATi experiments, recording vein pattern and morphological changes, across four dicot species with morphologically distinct types of compound leaves: pinnate tomato; bipinnate carrot; palmate lupin; and trifoliate clover. Results over the four morphologies corroborated the general effect of PATi on leaf complexity reported in tomato [23,29,30], *Cardamine* [22], and pea [31]. We also found a dosage dependence, with an overall stronger reduction in complexity at higher concentrations of PAT inhibitors. Previous work in tomato [23] and *Cardamine* [22] showed an effect of PATi on initial CP formation. We extend this to show how PATi affects vein development and vein network patterns in leaves and leaflets, across the diverse morphologies studied. The results are interpreted with the aid of a quantitative computer model of PAT and growth in the margin and lamina. The generation of some of the key experimental observations supports this as a conceptual framework for how auxin transport jointly mediates leaf complexity and venation in diverse types of compound leaves.

## 2. Results

### 2.1. Polar Auxin Transport Is Involved in Compound Leaflet Morphogenesis in Different Leaf Complexity Types

**Figure 1:**
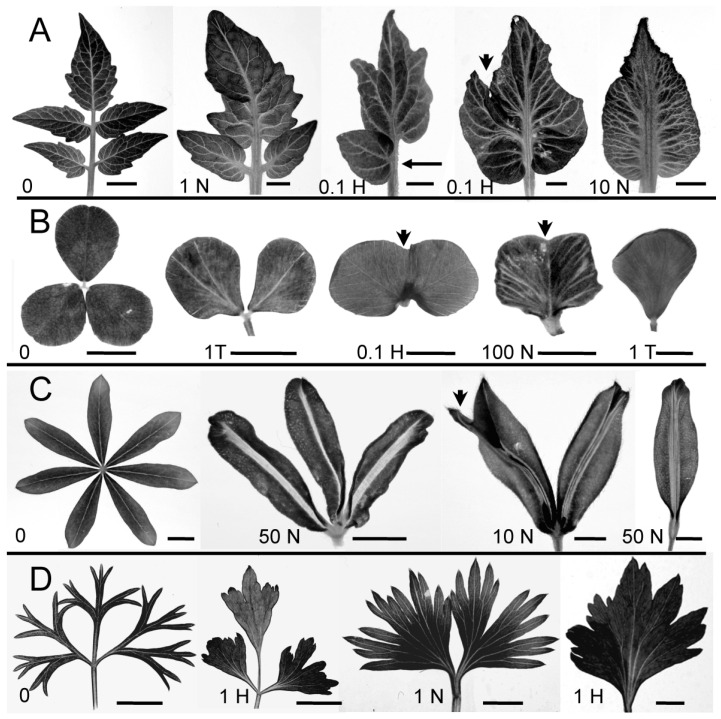
PAT inhibition reduced leaf complexity in ca 4 week old tomato plants ((**A**); leaf 3) and ca 2 weeks old clover ((**B**); leaf 2 and 3), lupin ((**C**); leaf 2 and 3), and carrot ((**D**); leaf 1 and 2). Images show control leaves (0) or leaves exposed to various concentrations (µM) of NPA (N), HFCA (H), or TIBA (T). The apparent absence of a lateral leaflet (arrow in (**A**)) and fusion between adjacent leaflets (arrowheads in (**A**–**C**)) are indicated. Scale bars are 5 mm ((**A**); control and NPA-treated), 2 mm ((**A**); HFCA-treated), or 0.5 mm ((**B**–**D**); except that (i) fourth image from left in (**B**) and (ii) third and fourth images from left in (**D**) are 0.2 mm).

Plants from the four different leaf complexity types were exposed to PAT inhibitors. While there was variability within species and treatment levels, all showed an overall reduction in leaf complexity, measured as leaflet count (Table 1, Figure 1). In general, leaf complexity decreased with increasing PAT inhibitor dosage. Different species and leaf numbers (L1, L2, L3) showed varying susceptibilities to different PAT inhibitors.

Tomato showed a significant mean change from the control leaflet count at NPA and HFCA concentrations as low as 0.1 µM. For all leaf numbers (L1–L3), an increased dosage of NPA or HFCA steadily reduced leaf complexity, from pinnately compound control leaves to simple leaves with a single blade (Figure 1A, L3 shown; Table 1); the simple leaf results were in greater contrast at L3 (which have higher leaflet count in control) than in L1 (lower count in control). For TIBA, only the highest tested concentration (10 µM) changed mean leaf complexity, and only in L2 and L3 (Table 1).

Clover and lupin likewise showed reduced leaf complexity with PATi. In clover, trifoliate control leaves showed reduction to two leaflets, one fused leaf with two laminar blades or a simple leaf depending on inhibitor concentration (Figure 1B). Significant reductions in mean leaflet number from the control were seen at the highest concentrations tested of HFCA and TIBA; while *p* > 0.1 for NPA, leaflet reduction may be indicated by the median leaflet count for L2 (Table 1). Lupin showed a significant change in leaflet count at the highest levels of NPA (Table 1; HFCA and TIBA not tested). The leaflet number decreased from palmately compound control leaves through simpler compound leaves to simple leaves (Figure 1C). In tomato, clover, and lupin, a decreased leaflet count appeared to occur either from inhibited leaflet formation (e.g., arrow in Figure 1A) or from the fusion of adjacent leaflets to form a single leaflet (e.g., arrowheads in Figure 1A–C).

Carrot showed a reduction from bipinnately compound to pinnately compound or even simple leaves with PAT inhibition (Figure 1D). While non-smooth margins complicated the quantification of the leaflet count and may have contributed to apparent increases in the mean leaflet number in some treatments, a significant reduction in mean complexity was seen with high NPA in L1 (Table 1).

### 2.2. Polar Auxin Transport Inhibitors Induced More Parallel Leaf/Leaflet Venation Patterning

Here, we use the terminology in [1] to describe venation patterning based on the positioning of the secondary veins relative to the single centrally localized primary vein in the leaf/leaflet lamina. Secondary vein types include secondaries that join with the primary and (i) run directly from the margin (craspedodromous), (ii) branch just within the margin, with one branch extending from the margin and the other joining with an adjacent secondary (semicraspedodromous), and (iii) joining with adjacent secondaries to form prominent arches (brochidodromous).

**Figure 2:**
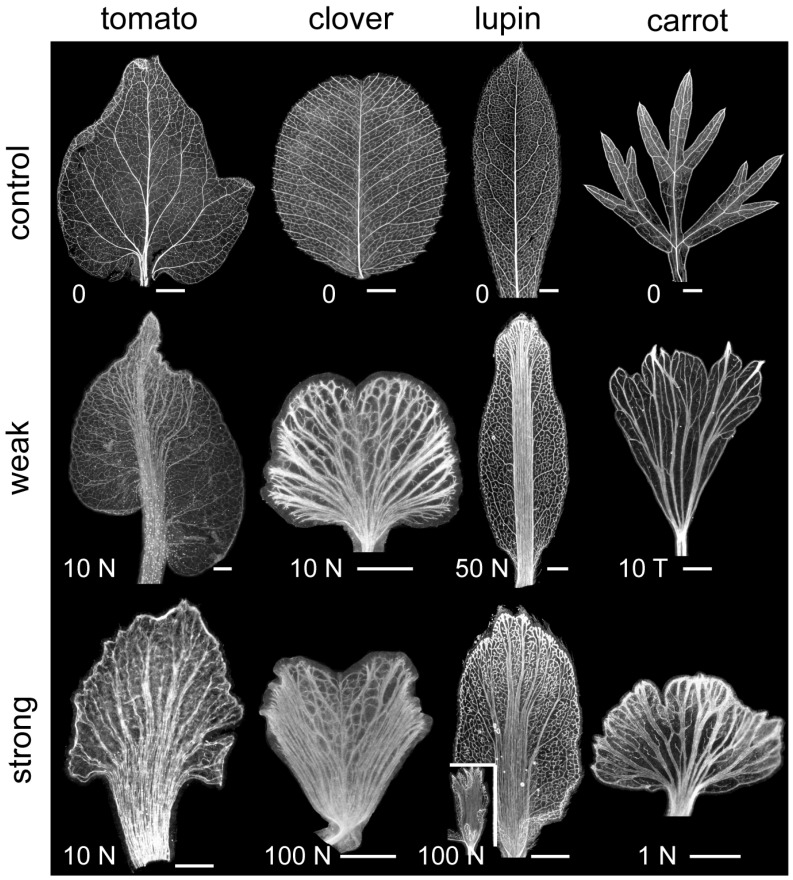
PAT inhibition induced more parallel-like leaf vein patterning. Dark-field images of tomato (Tiny Tom), clover, lupin, or carrot venation patterning on individual leaflets (control) or entire PAT-inhibited leaves (weak and strong phenotypes). Inset for lupin (100 N) shows very strong phenotype with parallel venation throughout lamina. Images show control leaves (0) or leaves exposed to various concentrations (µM) of NPA (N), HFCA (H) or TIBA (T). Scale bars are 1 mm.

All four species tested showed strong effects of PATi on vein patterning (Figure 2). These results indicate similarities in primary and secondary vein patterning in the leaflets of compound leaves to the PATi effects previously noted in simple leaves (e.g., [26]). PATi increased the number of secondary-like veins, with a distalization of the initiation points of these veins, such that the overall vein pattern tended to be more parallel to the proximodistal axis than in control. PATi interfered with the normal connection of veins, such as the secondary to primary connections in control, resulting in the supernumerary veins running in parallel bundles (see especially the central mid-leaflet bundles in PATi tomato and lupin, Figure 2).

Tomato leaflets have a semi-craspedodromous venation pattern, with secondary veins either joining adjacent secondaries or extending from the margin (e.g., Figure 1A and Figure 2). Control tomato leaflets had a hierarchical venation patterning with a central midvein, secondary veins forming loops alongside the midvein or extending from the marginal lobes towards the midvein, and higher-order marginal and areolar veins (Figure 1A and Figure 2). Weak PAT inhibition resulted in vascular overgrowth in the central lamina, appearing to involve the bundling of supernumerary secondary-like veins. In slightly stronger phenotypes, the distal region of the leaflet or leaf had a more diffuse region of multiple secondary-like veins that ran mostly parallel to the longitudinal axis, coalescing in more basal regions of the leaf/leaflet (Figure 2).

PAT inhibition had a similar effect on lupin leaf venation. Mature control lupin leaflets had hierarchical brochidodromous venation patterning with a primary midvein, secondary vein arches, and higher-order venation, whereas PAT inhibition resulted in more parallel venation in the center of the leaf/leaflet (Figure 2). Weaker PATi lupin phenotypes had a broad distal vein initiation region with multiple strands running parallel along the proximodistal axis, whereas stronger phenotypes had more secondary-like initiation sites at the apex that coalesced and bundled in the basal regions (Figure 2). Very strong PATi lupin phenotypes predominantly had parallel-type venation throughout the narrow lamina (Figure 2 inset).

Mature clover and carrot control leaves had a craspedodromous venation pattern—a primary midvein with secondary veins extending towards the marginal teeth (clover) or lobes (carrot) (Figure 2). Compared to control leaf patterns, PAT-inhibited clover and carrot leaves produced more parallel secondary veins, but with less distal coalescence and central bundling than observed in tomato and lupin, although carrot veins did coalesce near the petioles (Figure 2). All four species, with their different control venation patterns, showed a tendency towards more parallel secondary venation patterning with PAT inhibition.

**Figure 3:**
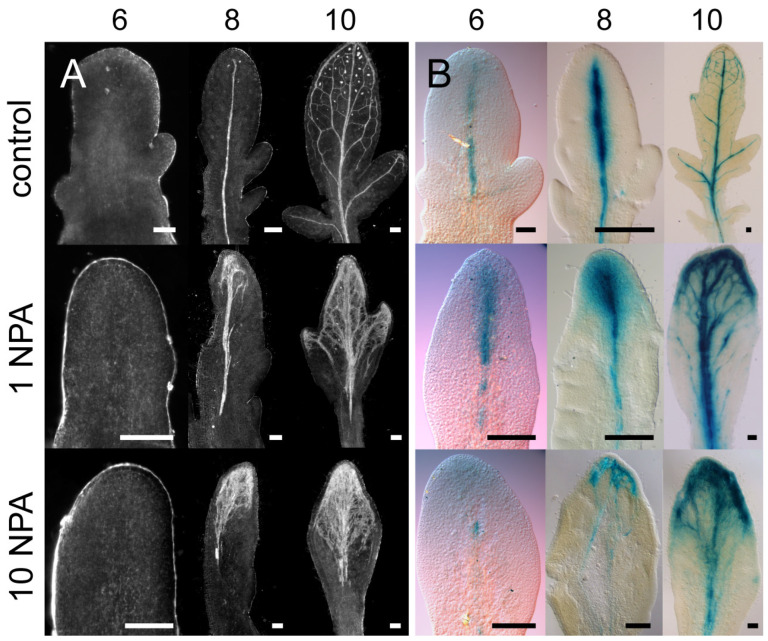
Leaf primordia venation and *pIAA2*::GUS expression in tomato (variety Alicia Craig). Tomato leaf primordia of plants grown for 6, 8, or 10 days in media with 0, 1, or 10 µM NPA. Primordia are viewed with darkfield optics to see differentiated venation patterns (**A**) or with brightfield optics to view *pIAA2*::GUS expression (**B**). NPA decreased leaflet outgrowth and induced vascular overgrowth and IAA2 accumulation near the leaf apex. (**A**) all 8 DAG primordia had a portion of the lamina cut off (left side of image) to assist visualization. Scale bars are 50 µm (6 DAG) or 100 µm (8 and 10 DAG).

**Figure 4:**
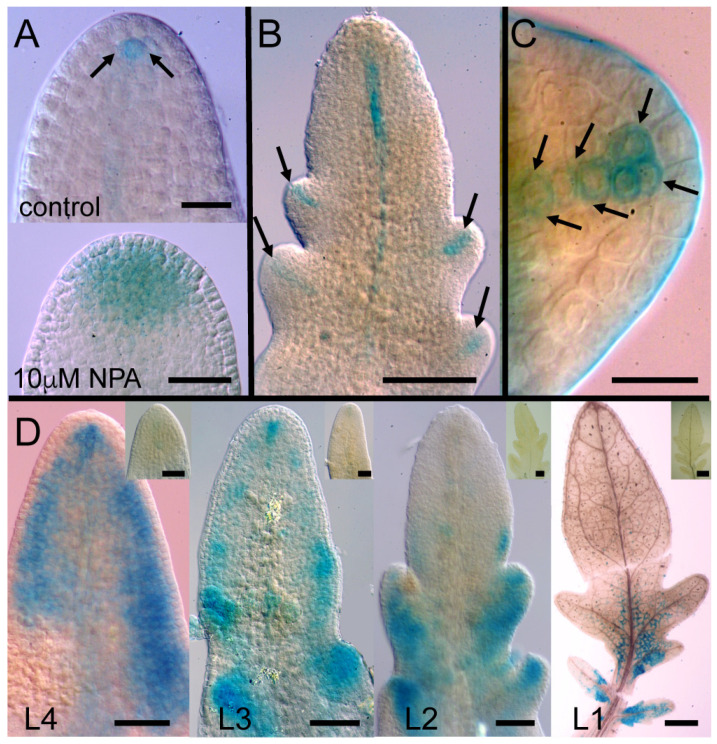
*pDR5*::GUS expression in tomato (variety Alicia Craig) leaf primordia. NPA treatment induced broad *pDR5*::GUS expression in the apex of 6 DAG L2 leaf primordia ((**A**), lower image) compared to the discrete auxin response maximum in control plants ((**A**), arrows in upper image). 7 DAG control L2 leaf primordia had discrete auxin response maxima in the emerging leaflets (arrows in (**B**)). Each developing leaflet had *pDR5*::GUS expression in a discrete auxin maxima below the epidermis as well as in the developing primary provascular cells of the midvein (arrows in (**C**)). Exposure of 8 DAG seedlings to 10 µM 2,4-D induced broad *pDR5*::GUS expression along most of the margin of L4 leaf primordia, with expression becoming restricted to more basal regions in older L3, L2, and L1 primordia ((**D**); insets show controls). Scale bars are 20 µm (**A**,**C**), 100 µm (**B**), or 50 µm ((**D**), except that L1 is 500 µm).

We examined the temporal development of leaflet shape and venation patterns in tomato. In control plants, the expansion of leaflets was evident by 6 DAG (days after germination), whereas NPA treatment inhibited leaflet outgrowth, especially at 10 µM NPA (Figure 3A). In control leaves, differentiated veins first became apparent along the leaf main axis (petiole/rachis), and then the semi-craspedodromous secondary venation of the terminal leaflet formed (Figure 3A). Eventually, differentiated midveins also extended along lateral leaflets to join the original leaf midvein (Figure 3A); these leaflets later also formed a semi-craspedodromous secondary venation pattern. 1 µM NPA treatment increased vascular development in the distal regions of the terminal leaflet and lateral leaflets as they emerged (Figure 3A). The supernumerary veins in PATi leaflets were poorly organized compared to control, filling the leaf lamina and showing decreased extension towards the proximal regions of the leaf. At 10 µM NPA, treated leaves had reduced complexity (lacked lateral leaflets) and showed increased distal initiation and a poorer extension of veins towards the leaf base compared to lower doses of NPA (Figure 3A). At maturity, 10 µM NPA-treated leaves showed simpler morphologies and supernumerary distally initiated, centrally-bundled veins, which could eventually connect to the rachis and/or petiole (Figure 2 and Figure 3A). Overall, these observations in veins corresponded to presumed auxin distributions as indicated by auxin response markers (Figure 3B and Figure 4A–C). In control plants, emerging leaf primordia and later also the lateral leaflets had discrete auxin maxima at their distal apex in the CPs (arrows in Figure 4A–C), as well as in procambial strands (Figure 3B and Figure 4B,C). In contrast, NPA treatment resulted in a large, diffuse auxin response zone at the emerging leaf apex and, if present, in lateral leaflet apices (Figure 4A).

### 2.3. A PAT-Growth Model Generates the Observed Vein and Morphological Responses to PATi

To aid in understanding these results, auxin transport and growth mechanisms were combined into a computer model. Simulations generating the observed reduction in leaf complexity and alterations in venation provide insight into how PATi induced both effects. Simulations correspond to the initial developmental sequence of the terminal and first lateral lobes and their midveins (such as in Figure 3A).

**Figure 5:**
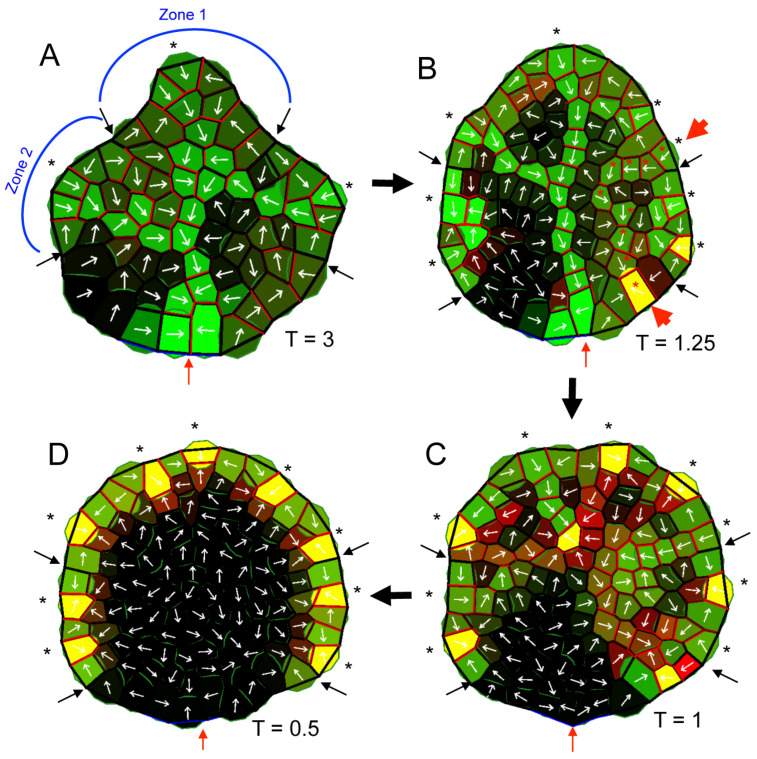
Computer simulations of the effect of PAT inhibitor treatment on leaf margin complexity. Images show the terminal lobe (Zone 1) and first two lateral lobes (Zone 2; only left side is marked in (**A**)), with black arrows delimiting the zone boundaries. Auxin-synthesizing cells in the margin grew in proportion to auxin concentration (green intensity). PIN1 concentration (red) was either cytosolic or in the membrane (shown at the walls). Yellow indicates high PIN1 and auxin concentrations within a cell; black indicates a lack of auxin and PIN1. White arrows indicate the net auxin flux for each cell. All results are shown at the same time (5 h 30 min computational units). PIN1-auxin dynamics in normal conditions (**A**) formed CPs (black asterisks) from which the midvein extended to the base of the leaf and the existing plant vasculature (represented by the blue-walled cells, red arrow) and from which secondary veins extended to the midvein. CPs are local auxin maxima in the margin (with marginal PIN1 polarization into the CPs), causing lobed outgrowth. As transmission through PIN1 (T parameter) was decreased (**B**–**D**), corresponding to increasing PAT inhibitor concentration, the leaf margin changed from lobed (**A**) to smooth (**B**–**D**), extra CPs formed (black asterisks), and secondary veins could run parallel to the midvein (red asterisks and red arrowheads in (**B**)). At low T (**C**), the mid-vein was lost, and the extra CPs extended short ‘strands’. At very low T (**D**), vein extension was lost. The smoother margins reflect the more uniform marginal auxin distributions (less localization to a few ‘discrete’ CPs) as PIN1 transmission was lost.

Modeling PAT inhibitor treatment as a decrease in auxin transmission through PIN1 (decreased *T* parameter) resulted in a smoothing of the leaf margin and loss of distinct sinuses (Figure 5 and Figure S1), corresponding to the experimentally observed reduction in leaf complexity (Figure 1). This was due to the effect of PATi on the spatial distribution of the auxin CPs, which catalyze cell growth at the lobe tips. In normal conditions, high auxin levels that were localized in the CPs induced an extension of distinct lobes. Reduced transport through PIN1 (corresponding to treatment with PAT inhibitors) impaired the normal auxin flow involved in maintaining a high auxin concentration in CPs and the spacing between CPs. With reduced flow, CPs drew auxin from fewer neighbouring cells, creating more CPs (black asterisks, Figure 5) that were more closely spaced than in normal conditions. The normal auxin distribution along the terminal and lateral margin in Figure 5A can be approximated by a sine wave with three peaks and a long wavelength separating fast growing lobes from slow growing sinuses; with reduced PAT (e.g., Figure 5D) the wavelength was shortened, with less space separating lobes and sinuses. This resulted in a more uniform auxin distribution than normal (i.e., local averages of auxin in PATi conditions tended to have a mix of high and low concentration cells, rather than either all high cells or all low cells as seen in control conditions). As auxin catalyzed growth, PATi produced more uniform leaf outgrowth, without the strong distinction between high and low growth rates between lobes and sinuses characteristic of complex leaves.

Altering PAT affected vein patterning in the lamina in addition to affecting CP spacing. Mild PAT inhibition (a reduction in the *T* transmissivity parameter) could produce secondary veins that run more parallel to the primary vein (red asterisks in Figure 5B and Figure S1F) rather than connecting to the primary vein (cf. Figure 5A). A further reduction in PAT (decrease in *T*) reduced vein extension (Figure 5C,D and Figure S1G–I), as seen experimentally (Figure 3). The reduced lobe extension in the lamina, loss of connection to the proximal sink (representing existing vasculature in the plant; red arrows, Figure 5), and impaired drainage of auxin leading to accumulation in the margin all correspond to experimental observations for auxin (Figure 3B) and vasculature (Figure 2 and Figure 3A). The model indicates that PAT dynamics and auxin-catalyzed growth can account for the simultaneous increase in vein number, altered lamina vein patterning, reduction in vein extension, marginal vein overgrowth, and loss of morphological complexity observed in PATi experiments.

### 2.4. Exogenous Auxin Application Altered Tomato Leaf Complexity but Not Venation Patterning

**Figure 6:**
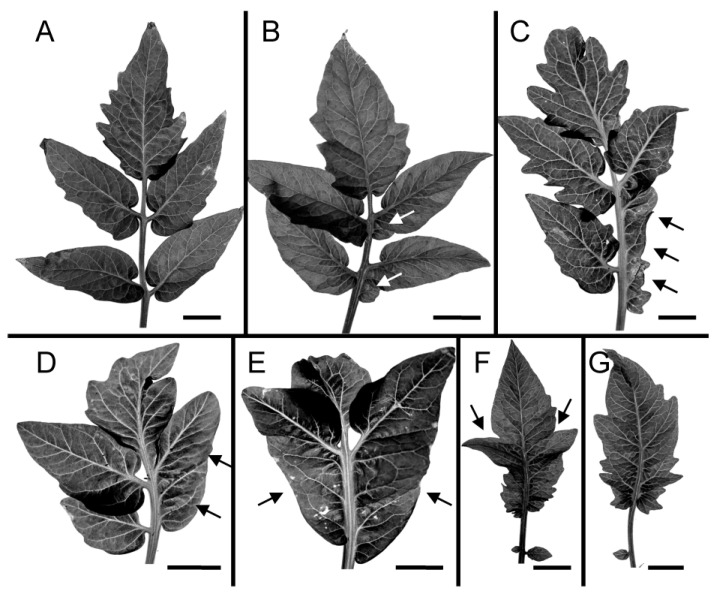
Exogenous IAA affected tomato (variety Tiny Tom) leaflet formation. Images show tomato leaves from ca 10-week-old control plants (**A**) or plants treated with 1% (**B**,**F**,**G**) or 10% (**C**–**E**) *w*/*w* IAA. IAA application resulted in leaves having broad bases fused along the petiole or rachis (arrows in (**B**,**C**) and/or fused with other leaflets (arrows in (**C**–**F**)). Scale bars are 10 mm.

Prior work reported cases of ectopic blade growth or extra leaflets with the local application of exogenous auxin application to tomato leaves [23]. We confirm this trend statistically: significant reductions in leaflet number with IAA application were found across three separate experiments (Table 2). In control plants, tomato leaflets always attached to the rachis via the short, cylindrical petiolule (Figure 6A). With IAA treatment, leaflets often had ectopic laminar growth on the petiole or rachis, which could extend along the rachis between leaflets (arrows in Figure 6B,C). Adjacent leaflets could be fused together, forming a single leaflet with broad attachments to the petiole/rachis (Figure 6D–F). Consequently, exogenous IAA treatment decreased the leaflet count (Figure 6D–F; Table 2). In some cases, the decreased leaflet count may have been the result of inhibited leaflet formation rather than a fusion of adjacent leaflets (Figure 6G). In general, 1% *w*/*w* IAA treatment resulted in less severe perturbations in growth, with leaflets often forming broad bases of attachment to the petiole or rachis rather than fusions of adjacent leaflets. Thus, plants treated with 1% *w*/*w* IAA generally had more leaflets than plants treated with 10% *w*/*w* IAA (Table 2).

Nevertheless, both 1% and 10% *w*/*w* IAA treatments resulted in substantial percentages of leaves with leaflets ectopically fused along the petiole or rachis and/or to each other (Table 2). While fusions were never observed in control leaves, fusions were observed frequently in treatments with exogenous IAA (Table 2). In general, younger leaf primordia (higher leaf number) showed a stronger effect from IAA treatment (Table 2; particularly Tiny Tom 2), with the formation of callus-like structures or a complete inhibition of growth in very young leaf primordia exposed to exogenous IAA. A similar tendency towards the fusion of the leaflets to the petiole/rachis and/or to adjacent leaflets with IAA treatment was observed in the tomato variety Glamour (Table 2; Figure S2). These observations indicate that exogenous IAA application disrupted the boundaries delimiting lobes in developing tomato leaflets.

The exposure of 8 DAG seedling leaves to the synthetic auxin analog 2,4-D induced broad ectopic auxin response maxima (Figure 4D), corresponding to the lobe broadening observed for exogenous IAA (Figure 6). In young L4 leaf primordia exposed to 2,4-D, *pDR5*: GUS expression was observed particularly at the margins of the lamina, whereas expression was progressively restricted to more basal regions in older L3, L2, and L1 leaves (Figure 4D). This corresponds to the smaller effect on complexity in older leaves with exogenous IAA, particularly for the percentage of leaves with fused leaflets in IAA Experiment 2 (Tiny Tom 2; Table 2). Little or no *pDR5*:GUS expression was observed in control leaves grown in liquid media at this developmental stage (insets, Figure 4D).

**Figure 7:**
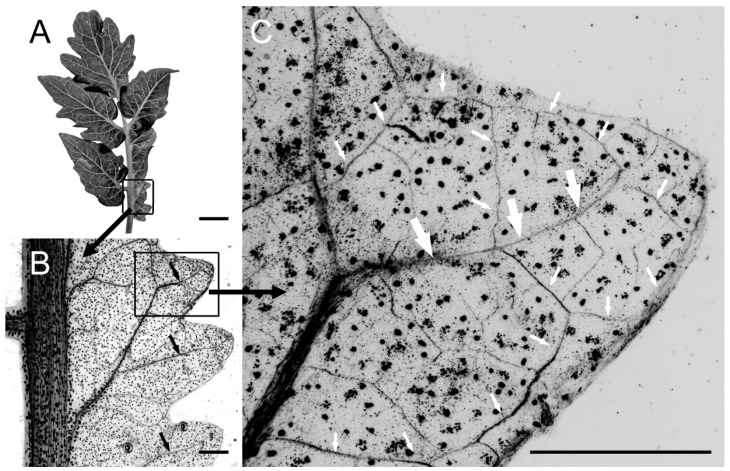
IAA treatment did not alter overall tomato leaf venation patterning. Magnified images (**B**,**C**) of a leaf (**A**) treated with 10% *w*/*w* IAA. Darkfield images were inverted to show leaf venation patterning, and numerous starch granules are also present. IAA treatment induced primary-like (arrows in (**B**); large arrows in (**C**)) veins extending towards the lobe apex and secondary-like (small arrows in (**C**)) vein loops. Scale bars are 10 mm (**A**) or 1 mm (**B**,**C**).

Because the application of exogenous IAA reduced developing complex tomato leaves to more simple leafed structures (Figure 6; Table 2), similar to the morphological effect with PAT inhibition, we wanted to determine whether the semi-craspedodromous venation patterning was also simplified to more parallel-like venation. This was not the case. Exogenous IAA application to developing tomato leaves retained normal venation patterning in regions of ectopic laminar growth and/or laminar fusion between leaflets (Figure 7). Secondary veins extended from the ectopic laminar region into the midvein, joining with other secondary veins to form loops. Additional veins branched from the marginal lobes towards these secondary loops. Each marginal lobe had a semi-craspedodromous venation patterning, with a primary-like midvein extending up to or near the lobe tip, secondary-like vein loops on either side, and higher order venation (Figure 7). Hence, unlike PAT inhibition, a reduction in tomato leaf complexity with exogenous IAA was not correlated with the induction of parallel-like venation patterning.

### 2.5. Modeling Suggests That Vein Developmental Stage Affects the Different Morphological and Venation Responses to Exogenous IAA Exposure

**Figure 8:**
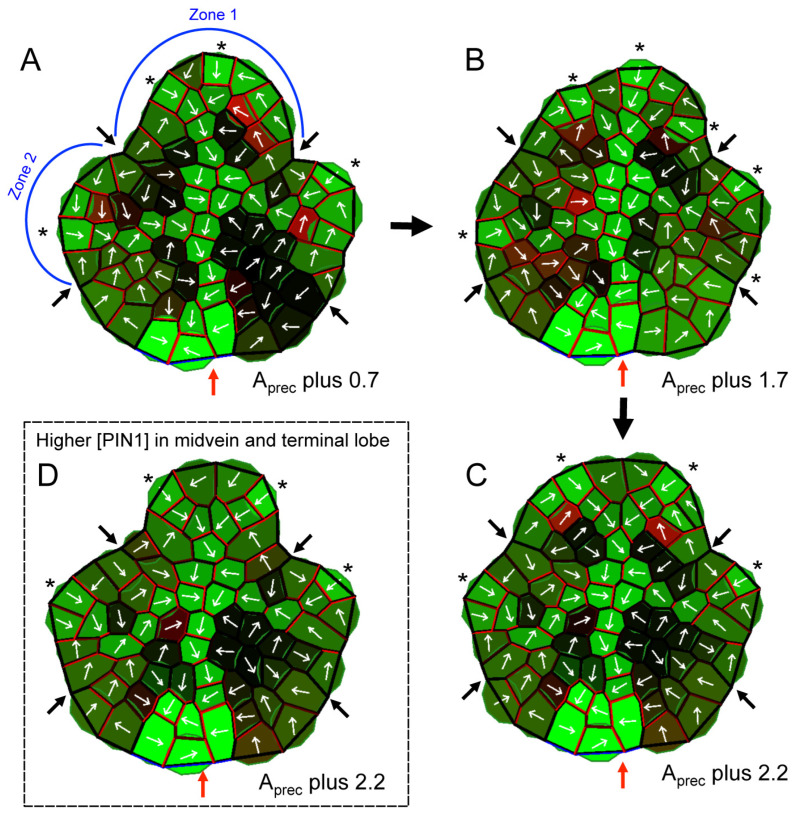
Computer simulations of the effect of exogenous IAA on leaf margin complexity. Model parameters and visualization are as in Figure 5. (**A**–**C**) Increasing auxin production, corresponding to exogenous IAA application, produced smoother margins (auxin precursor concentration, A_prec_, was boosted at the time the lateral lobes became auxin-synthesizing). Larger boosts created broader lobes and shallower clefts: (**A**) 0.7 added to A_prec_; (**B**) 1.7 added to A_prec_; (**C**) 2.2 added to A_prec_. (**A**–**C**) Transport from the margin was not sufficient to reduce auxin levels to normal, resulting in the extra growth of the lobes. (**D**) The leaflets had the same A_prec_ boost as (**C**), but PIN1 was also boosted in the terminal lobe and midvein to simulate established vasculature in more mature leaflets. In this case, the high doses of exogenous auxin could be sufficiently drained, resulting in a narrower, more normal lobe shape (compare region between black arrows delimiting zone 1 in (**C**,**D**)). Asterisks indicate CPs.

To understand this different response in terms of the PAT mechanism, we simulated exogenous IAA application by a boost of the auxin precursor level, A_prec_, parameter in the computer model. This increased the marginal auxin supply in specific zones, which catalyzed the additional growth of the lobes. As auxin was higher throughout the lobes and not as focused in the CPs, this produced broader lobes and less distinct sinuses than normal (Figure 8), corresponding to a reduction in complexity. Consistent with the concentration effects in Table 2, higher A_prec_ (corresponding to a higher added IAA concentration) produced broader lobes (Figure 8A–C). Different effects of exogenous IAA by leaf age (L-stage, Table 2) may reflect the maturity of the vasculature: in Figure 8D, PIN1 is boosted in the terminal margin and midvein (to 150 from normal values of up to 120) to represent more mature vasculature (localized PIN1 is observed to increase in veins over the course of development, e.g., [14,15,24]. In this case, the same added auxin is more efficiently drained away and does not affect morphology as much as in the terminal lobe of Figure 8C (normal PIN1 levels, representing the less developed vasculature of a younger leaf). Hence, exposure to increased IAA in leaves with less mature vasculature resulted in a broader terminal lobe with less distinct sinuses (Figure 8C) compared to a leaf with more mature vascular cells capable of transporting auxin away from the margin (Figure 8D).

While the above results suggest a role of vascular maturation (and hence auxin transport capabilities) on lobing, altering leaf morphology with exogenous IAA had no effect on vascular patterning. Models with normal levels of transmission through PIN1 showed that added auxin may spur marginal growth and lobe broadening, but PAT flows remained sufficient to maintain normal CPs and a midvein in each lobe (Figure 8A–C). Modelling suggests that the difference in results between PATi and exogenous IAA application stems from different aspects of the PAT-growth mechanism. The relative independence of morphogenesis and vein patterns with IAA application reflect that while added auxin may catalyze growth, it was also efficiently transported by normal PIN1 function and did not alter vein patterns (Figure 8). In contrast, PATi-impeded auxin flow shortened CP–CP distances, multiplying the number of CPs and veins, in addition to altering marginal growth and therefore lobing (Figure 5).

## 3. Discussion

### 3.1. PATi Reduced Complexity in Diverse Compound Leaf Types

We chose species with a variety of leaf complexity types. PATi could reduce compound leaves as diverse as tomato, with deep serrations and leaflets, to the repeated bifurcations of carrot, to the radially arranged and completely separated leaflets of lupin and clover to simple leaves. Both leaf shapes (Figure 1) and vein patterns (Figure 2) were affected, indicating a role for auxin transport in these processes. Different responses to PATi and to exogenous IAA indicate features of the auxin patterning mechanism involved in morphogenesis and venation. The common general response across these species indicates the role of auxin in compound leaves is at least some 120 million years old, when the Asterids (tomato and carrot) and Rosids (clover and lupin) diverged [32]. Aspects of the PIN–auxin module are likely even more ancient, appearing in mosses [33].

Differences in the species’ sensitivities to PATi agents and dosage (Table 1) indicate between-species differences in auxin transport that have arisen more recently. These differences may involve a differing inhibitor affinity for binding sites between species. The crystal structure for the *A. thaliana* PIN1 protein has been resolved, and it is known that NPA competes with and binds to a similar position as the natural auxin IAA [27]. Different responses to different NPA dosages in other species may therefore indicate the affinity of IAA binding to PIN1 relative to *Arabidopsis*. Response strengths to other PAT inhibitors may also indicate varying binding strengths relative to the *Arabidopsis* IAA/NPA sites.

### 3.2. PATi Produced Supernumary Veins with More Parallel Patterning

The four species studied also represent different vein pattern types: craspedodromous (clover and carrot); semi-craspedodromous (tomato); and brochidodromous (lupin). One of the main distinguishing features of leaf venation is the positioning of the primary and secondary strands with respect to the main leaf axis and to each other. Craspedodromous and brochidodromous secondary veins join with the central primary vein throughout the leaf lamina. PATi affected both the number of secondaries and their angle of approach towards the central lamina.

In the four species tested, PATi increased the number of secondary veins, reflecting the increased number of marginal CP sites (Figure 2). These responses are similar to those observed in *Arabidopsis* simple leaves grown in mild to moderate PATi conditions [25,26]. In *Arabidopsis*, very strong PAT inhibition often results in more vascular tissue around the margins where auxin pools, forming thicker (or bundled) veins with retarded vein extension from the margin towards the leaf base [25,26]. PATi also retarded vein extension in tomato leaf primordia (Figure 3A), although they did eventually extend into the basal lamina by leaf maturation (Figure 2). PATi caused less marginal vascularization than in *Arabidopsis*, possibly because under our experimental conditions these species may have retained enough vascular function to effectively drain auxin from the margins, thus preventing excess marginal vascularization. All four species did show an increased thickness (or bundling) of the secondary veins with PATi (Figure 2), similar to *Arabidopsis*.

PATi in all four species usually eliminated the single dominant midvein (primary vein) of the leaf/leaflet, producing more parallel venation patterning, with secondary-like veins running more parallel to the proximodistal leaf/leaflet axis (Figure 2). There appear to be two aspects to this parallelization: (a) the marginal sources of the secondaries (CPs) appear to become more distal with PATi compared to control; and (b) PATi appears to enhance proximal-wards bending (i.e., a decreased angle of approach) in secondaries as they approach the central lamina, producing parallel bundles as compared to the secondary to primary vein connections observed in controls. The marginal auxin sources of secondaries appeared more distally focused in tomato and lupin, producing bundling along the length of the leaf/leaflet center. In contrast, auxin source distalization was broader and less centralized in clover and carrot, with the leaf/leaflet vascular bending and bundling towards the center occurring more proximally, near the petiole. The angle of approach of secondaries to the midvein region may suggest that PATi interferes with the normal connection process of veins (bundling is also observed in more marginal positions, in the thickening or bundling of secondaries with PATi). Potentially, as the attraction or ability to connect to established veins (such as secondary to primary) is decreased, the increased relative attraction of the leaf base (older venation within the stem) induced the proximal-wards turn in the secondaries.

The diminished primary vein strength with *Arabidopsis* simple leaves similarly shows some of these two parallelization aspects under PATi [25,26]. Compared to the compound leaf types studied here, *Arabidopsis* simple leaves tend to exhibit broader auxin sources (less CP distalization) and more vein bending. The variety of source distalization and vein bending of secondaries observed in Figure 2 may indicate that different constraints on PAT may exist in different compound leaf geometries or developmental programs.

### 3.3. The Effects of Exogenous IAA Varies with Developmental Stage and Can Differ from PATi

The application of exogenous IAA to developing tomato leaf primordia disrupted leaflet boundary formation, resulting in ectopic laminar growth along the rachis/petiole and fusion between adjacent leaflets that also reduced overall leaf complexity (Figure 6, Table 2). The competency to respond to exogenous IAA depended on the developmental stage. In tomato, auxin response domains were observed throughout the margin of very young leaf primordia and only in more basal (younger) regions of older leaf primordia (Figure 4D). Modeling suggests that exogenous IAA does not impact the morphology of older lobes with more mature veins that can efficiently transport the excess IAA away from the margin (Figure 8). This would explain why fused leaflets were mostly observed between more basal (younger) leaflets if auxin was applied to older leaf primordia (e.g., Figure 6B–E), and the extent of fusion was greater in more newly formed leaves (particularly Experiment 2, Table 2). These younger regions had immature veins that did not remove the excess IAA from the margin during leaf shape formation, resulting in diffuse marginal auxin and leaflet fusion.

While exogenous IAA application can alter the normal pattern of discrete auxin maxima in the margin, particularly in new leaf primordia (Figure 4D), vein patterning appeared largely unaffected in mature leaves even in fused regions (Figure 7). Normal vein patterning suggests that, even in the presence of exogenous IAA, functional veins were eventually formed that could effectively transport the excess IAA from the margin, such that vein patterning was not altered. With PATi, auxin pooled at the margin because it disrupted vein formation and their auxin transport efficiency, creating more marginal CPs and hence supernumerary veins. This suggests that the PATi induction of supernumerary more parallel venation is the result of reduced PAT and not just the altered auxin levels in the margin.

### 3.4. A PAT-Growth Model for the Morphological and Venation Effects of PATi and Exogenous IAA

The mathematical model of PAT and auxin-dependent growth dynamics captures key features of the experimental results. These include: the proliferation of CPs and generation of supernumerary veins with PATi, with the corresponding reduction in morphological complexity (Figure 5); and the morphological effects of exogenous IAA, which can occur without alterations of the venation patterns (Figure 8).

Modeling indicates that the different venation responses of PATi and exogenous IAA could be due to differences in vein development. Both cases had higher than normal auxin levels in the margin, leading to the formation of more CPs with fewer cells feeding auxin into them. PATi-induced PIN1 disruption affected early plastic stages of vascular development, as the marginal auxin levels remained high, thereby creating more CPs that drive the formation of new secondary veins. With exogenous IAA, veins can still develop normally (as PIN1 localization was not affected) and were able to mitigate the excess auxin in the margin, such that venation patterns were not significantly altered. Vein maturation status at the time of exogenous IAA exposure may also play a role—older leaf primordia / lobes with more mature vascular cells were able to drain auxin more efficiently and therefore had less growth defects compared to younger leaves/lobes with less developed vasculature (e.g., Figure 8D compared to Figure 8C).

The similar effects of lobe broadening for PATi or IAA boosting may occur for different reasons. PATi reduced auxin drainage, resulting in higher auxin in the margin. This created supernumerary CPs that caused more spatially averaged auxin distribution and more uniform marginal growth because the auxin was not being drained away. Exogenous IAA boosted overall marginal auxin in the lobe, lessening the relative high concentration/localization of the CP at the lobe tip, leading to more uniform marginal growth and additional CPs with exogenous IAA.

Previous models included the CP formation and growth of the margin [9,13,34] but did not compute PAT in the lamina of the leaf. By modeling PAT for the margin and the interior of the leaf, our model can address the more complete picture of how PATi interferes with the normal ‘reverse fountain’ patterning in which PAT plays a role in both CP formation and flow at the margin, as well as in the inward extension and canalization of provascular auxin traces. In particular, this allows the model to capture the effects of auxin drainage from the margin sources, key to interpreting the results of the exogenous IAA experiments. For PATi, the model generates the observed increase in CPs corresponding to supernumerary veins.

We note that our model only has uniform and auxin-dependent growth. The inclusion of additional factors, such as CUC pattern restraining growth in sinuses [9,34], would be expected to enhance lobe–sinus differences and support clearer boundaries in the resulting morphologies. Questions remain regarding the causes of the venation effects of (a) more distalized marginal sources and (b) secondary vein turning rather than joining prior veins (see discussion above) under PATi. Modeling could further explore (a) the PAT influence of marginal auxin synthesis zones and (b) mechanisms for turning the secondaries in the mid-leaf. The model does not presently include (a), but a slight turning (b) was achieved (Figure 5B). As discussed in [35], vein turning could be due to PATi effects on the up-the-gradient (UTG) allocation of PIN1 needed to connect an extending (secondary) vein to an established (primary) vein with high auxin. In this case, the different degrees of vein turning observed between the present four species could represent different degrees of the role of UTG in vein connection across these species. It is likely, though, that other genetic or transport factors (such as plasmodesmata, [36,37]) also influence vein connections and would be needed for a more complete description of PATi effects on vein bending.

## 4. Materials and Methods

### 4.1. Plant Material and Growth Conditions

Four plant species were used: (1) tomato, *Lycopersicon esculentum* L. (varieties Glamour and Tiny Tom, Stokes Seeds Ltd., St. Catharines, ON, Canada; PATi experiments on Glamour, IAA experiments on both varieties); (2) white clover, *Trifolium repens* L. (Dawson Seed Co. Ltd., Surrey, BC, Canada); (3) carrot, *Daucus carota* (variety Crona; Svalöv/Weibull AB, Svalöv, Sweden); and (4) lupin, *Lupinus polyphyllus* (wild seed collected from Burnaby Mountain Park, Burnaby, BC, Canada).

PAT inhibitors were used on all four species. Seeds were rinsed in water for about 30 min to remove commercial fungicides (where applicable), sterilized for 1 min in 70% *v*/*v* ethanol and then 10 min in 50% *v*/*v* commercial bleach, followed by three rinses in sterile water. Lupin seeds were scarified prior to sterilization. Seeds were plated on solid ATS medium [38] supplemented with either N-(1-Naphthyl) phthalamic acid (NPA; TCI, Tokyo, Japan), 9-hydroxyfluorene-9-carboxylic acid (HFCA; Sigma, St. Louis, MO, USA), or 2,3,5-triiodobenzoic acid (TIBA; Sigma, St. Louis, MO, USA) dissolved in DMSO to a final concentration of 0.1, 1, 10, 50 or 100 µM, or an equivalent volume of DMSO for control material. Seeds were plated in 9 cm diameter Petri dishes (ca 20 seeds/plate), stratified at 4 °C overnight, and then grown at about 20 °C, 16 h illumination (ca 150 to 200 µmol m^−2^ s^−1^) for 2 weeks. At this time, seedlings were transferred to larger containers with sterile media and grown for another 2 to 3 weeks. For analyses of the early development of tomato leaf primordia, sterilized seeds were plated (ca 10 seeds/plate) in 9 cm Petri dishes containing 10 mL of liquid ATS (control, or supplemented with NPA to final volumes of 1 or 10 µM), stratified at 4 °C overnight, and then gently agitated at ca 20 °C with constant illumination for up to 10 d growth.

The effect of exogenous indole acetic acid (IAA) was examined in tomato. Seeds were immersed in water and stratified at 4 °C overnight and then planted in soil (Pro-Mix general purpose, Premier Horticulture Inc., Quakertown, PA, USA) and grown in a greenhouse. Three experiments were performed where IAA (Sigma, St. Louis, MO, USA) was mixed with lanolin at 0, 1, or 10% *w*/*w* concentrations and a small drop placed at the base of the following leaf primordia: (i) leaf 2 (L2) and L3 in 2-week-old Tiny Tom plants (Experiment 1); (ii) L5 and L6 in 6-week-old Tiny Tom plants (Experiment 2); (iii) L4 and L5 in 4-week-old Glamour plants (Experiment 3). Leaf complexity and venation patterning were assessed in mature leaves: L2 to L5 in 6-week-old plants (Experiment 1); L4 to L7 leaves in 10-week-old plants (Experiments 2, 3).

Two auxin responsive markers, *pDR5*::GUS (obtained from G. Hagen, University of Missouri, Columbia, MO, USA; [39]) and *pAtIAA2*::GUS (gift from J. Normanly, University of Massachusetts, Amherst, MA, USA; [40]), were introduced into tomato (variety Ailsa Craig) by *Agrobacterium*-mediated transformation [41]. To test the effect of auxin transport inhibition on auxin response maxima, transformed tomato plants were grown as above in solid ATS or ½ Murashige Skoog media supplemented with 0, 1, or 10 µM NPA, and leaves 1 and 2 excised at 6, 8, and 10 d growth. Leaves were stained for GUS expression as in [25] or using a modified GUS staining solution (100 mM phosphate buffer, 5 mM K_3_(Fe(CN_6_)), 5 mM K_4_(Fe(CN_6_)), 10 mM EDTA, 10% *v*/*v* methanol, 0.01% *v*/*v* Triton X 100, 5 mM X-GLUC (Gold Biotechnology Inc., St. Louis, MO, USA)). To determine the competency of cellular response to exogenous auxin, *pDR5*::GUS seedlings were grown for 8 days in liquid ATS medium, and then excised leaves were transferred to new media containing 0 or 10 µM 2,4-D (Sigma, St. Louis, MO, USA), agitated gently for 16 h, and stained for GUS expression as in [25].

### 4.2. Analyses of Leaf Complexity and Venation Patterning

We use the terminology from [1] to describe leaf complexity types and leaf venation patterns. To examine the effect of exogenous auxin on tomato leaf complexity, the number of leaflets was counted, and the extent of fusion of leaflets along the rachis and/or to each other was scored 6 weeks after the application of exogenous IAA to leaf primordia. To examine the impact of auxin transport inhibition on leaf complexity, the number of leaflets (for tomato, white clover, carrot, and lupin) was counted for the first three mature leaves (L1, L2, L3) of 4- to 5-week-old seedlings grown on solid media with or without auxin transport inhibitors. Selected mature leaves of all species grown with or without inhibitors were also cleared and analyzed for vascular patterning as in [26]. For tomato seedlings grown in liquid media, developing L1 and L2 leaf primordia in 6–10-day-old seedlings were cleared, and their complexity and vascular patterning analyzed as above. Images were taken using a Nikon Eclipse E600 microscope (Nikon Instruments Inc., Tokyo, Japan) and a Canon EOS D30 digital camera (Oita Canon Inc., Oita, Japan).

### 4.3. Computer Simulations of Leaf Venation and Growth

Computer simulations of PIN1 and auxin dynamics used the model and parameters described in [35], written within the VirtualLeaf software Version 1.0.2 package [42]. This dual-polarization model produces both CPs and inward-extending provascular tracks (see also [24,43]). Installation, files, and parameter values are discussed further in Supplementary Text S1.

The generation of the normal sequence of a primary (distally localized) auxin maximum with the subsequent formation of secondary (more proximal) auxin maxima was coupled to auxin-dependent cell growth. This generated a multi-lobed leaf shape (with a central terminal lobe and two lateral lobes), representing a compound leaf.

Auxin synthesis occurred from a precursor in the margin, A_prec_. (See Introduction of [35]: the computational margin represents the epidermal and subepidermal cells in which auxin is synthesized and CPs form.) The earlier development of the terminal lobe was represented by a higher initial A_prec_ value than in the lateral lobes. A_prec_ increased in all cells to a maximum. Leaf growth was generated by increasing margin cell areas according to background and auxin-dependent rates, followed by cell division. PAT inhibitor treatment was simulated by decreasing the transmission of auxin through PIN1 (*T* parameter). Exogenous IAA treatment was simulated by increasing A_prec_ a given amount in all auxin-synthesizing cells at 2 h 47 min (computational units), when the lateral lobes become auxin-synthesizing.

## Figures and Tables

**Table 1:** Polar auxin transport inhibitors reduced leaflet count. Leaflet count for the first three mature leaves (L1, L2, L3) at circa 4 weeks in control conditions or exposed to different concentrations (µM) of NPA (N), HFCA (H), or TIBA (T). Cells show: Row 1 = mean ± S.D. and (n); Row 2 = *p*-value for mean treated value being equal to control, * significantly different from control (*p* < 5 × 10^−2^) or ** highly significantly different from control (*p* < 1 × 10^−2^); Row 3 = % below control median, note increase in percentage with treatment. Colors show: Green = treatment sample mean below control; White = no difference; Orange = treatment sample mean above control. ND = not done. Undefined = *p*-value undefined due to zero S.D.

Plant	Leaf	Control	0.1 H	1 H	0.1 N	1 N	10 N	0.1 T	1 T	10 T
	L1	2.8 ± 0.6 (21)	2.2 ± 0.8 (18)	1.1 ± 0.3 (9)	1.9 ± 0.7 (17)	1.3 ± 0.6 (22)	1.0 ± 0 (17)	2.7 ± 0.8 (15)	3.0 ± 0.6 (13)	2.0 ± 0.9 (6)
	*p*-value		8.0 × 10^−3^ **	3.0 × 10^−10^ **	1.4 × 10^−4^ **	1.1 × 10^−9^ **	1.1 × 10^−11^ **	7.6 × 10^−1^	3.7 × 10^−1^	7.9 × 10^−2^
	% with < median	19	61	100	82	91	100	20	15	67
	L2	3.5 ± 0.8 (22)	2.5 ± 1.1 (17)	1.1 ± 0.3 (11)	2.7 ± 0.6 (17)	1.8 ± 0.9 (22)	1.0 ± 0 (17)	3.6 ± 0.9 (14)	3.3 ± 0.6 (14)	2.3 ± 1.0 (6)
tomato	*p*-value		4.9 × 10^−3^ **	4.3 × 10^−13^ **	1.8 × 10^−3^ **	4.4 × 10^−8^**	2.4 × 10^−12^ **	7.0 × 10^−1^	4.8 × 10^−1^	4.4 × 10^−2^ *
	% with < median	5	47	100	24	73	100	7	0	33
	L3	4.5 ± 0.9 (22)	2.9 ± 0.9 (18)	1.5 ± 0.5 (6)	2.9 ± 0.6 (16)	2.1 ± 1.0 (22)	1.0 ± 0 (14)	4.5 ± 1.0 (14)	4.0 ± 1.0 (13)	3.2 ± 0.4 (6)
	*p*-value		2.5 × 10^−6^ **	1.8 × 10^−7^ **	1.4 × 10^−7^ **	1.1 × 10^−10^ **	1.2 × 10^−14^ **	7.1 × 10^−1^	1.4 × 10^−1^	7.5 × 10^−3^ *
	% with < median	32	94	100	100	100	100	21	62	100
	L2	2.8 ± 0.6 (13)	2.9 ± 0.3 (9)	1.6 ± 0.5 (7)		5 ± 0 (5)	2.0 ± 0.8 (4)	2.7 ± 0.6 (3)	1.8 ± 0.5 (4)	
	*p*-value		8.2 × 10^−1^	2.4 × 10^−4^ **	ND	8.5 × 10^−9^ **	1.3 × 10^−1^	6.6 × 10^−1^	1.1 × 10^−2^ *	ND
clover	% with < median	8	11	100		0	75	33	100	
	L3	3.0 ± 0 (13)	2.4 ± 0.7 (9)	1.9 ± 1.0 (8)		3.0 ± 0 (5)	2.5 ± 0.8 (6)	3.0 ± 0 (3)	2.3 ± 1.0 (4)	
	*p*-value		5.1 × 10^−2^	1.5 × 10^−2^ *	ND	undefined	2.0 × 10^−1^	undefined	2.1 × 10^−1^	ND
	% with < median	0	44	63		0	33	0	50	
	L2	6.6 ± 0.8 (47)				6.3 ± 0.6 (3)	4.0 ± 2.3 (57)			
	*p*-value		ND	ND	ND	4.9 × 10^−1^	1.4 × 10^−11^ **	ND	ND	ND
lupin	% with < median	45				67	86			
	L3	7.0 ± 0.7 (48)				6.3 ± 0.6 (3)	5.3 ± 2.4 (55)			
	*p*-value		ND	ND	ND	1.7 × 10^−1^	5.2 × 10^−6^ **	ND	ND	ND
	% with < median	25				67	69			
	L1	5.6 ± 1.2 (17)	5.2 ± 0.6 (13)	4.2 ± 2.1 (8)	5.4 ± 0.9 (5)	1.7 ± 1.6 (6)		5.4 ± 1.3 (9)	5.0 ± 0 (9)	
	*p*-value		1.9 × 10^−1^	1.3 × 10^−1^	7.1 × 10^−1^	1.0 × 10^−3^ **	ND	7.9 × 10^−1^	5.6 × 10^−2^	ND
	% with < median	6	0	25	0	83		0	0	
	L2	6.4 ± 1.4 (14)	7.9 ± 1.0 (11)	6.4 ± 2.5 (7)	6.6 ± 1.7 (5)	5.8 ± 1.8 (5)		7.5 ± 1.4 (8)	7.0 ± 1.2 (7)	
carrot	*p*-value		4.9 × 10^−3^ **	9.5 × 10^−1^	7.8 × 10^−1^	5.5 × 10^−1^	ND	9.1 × 10^−2^	2.9 × 10^−1^	ND
	% with < median	50	0	29	40	40		13	14	
	L3	6.9 ± 1.1 (12)	9.0 ± 1.0 (9)	7.4 ± 2.6 (5)				8.0 ± 1.1 (6)	8.6 ± 0.9 (5)	
	*p*-value		2.4 × 10^−4^ **	7.1 × 10^−1^	ND	ND	ND	7.5 × 10^−2^	8.8 × 10^−3^ **	ND
	% with < median	17	0	20				0	0	

**Table 2:** Exogenous IAA application reduced leaflet count in tomato. Leaflet number in control plants or after the addition of IAA. Cells show: Row 1 = mean ± S.D. and (n); Row 2 = *p*-value for mean treated value being equal to control, * significantly different from control (*p* < 5 × 10^−2^) or ** highly significantly different from control (*p* < 1 × 10^−2^); Row 3 = % below control median, note increase in percentage with treatment; Row 4 = % of plants with fused leaflets. ND = not done. Tiny Tom 1, 2 = experiment 1 or 2 with Tiny Tom. G = Glamour variety.

Leaf	TT 1; Control	TT 1; 10% IAA	TT 2; Control	TT 2; 1% IAA	TT 2; 10% IAA	G; Control	G; 10% IAA
Leaf 2	3.5 ± 0.8 (48)	3.1 ± 1.1 (59)					
*p*-value		1.5 × 10^−2^ *	ND	ND	ND	ND	ND
% < median	6	22					
% fused	0	20					
Leaf 3	4.5 ± 0.9 (48)	3.4 ± 1.3 (59)					
*p*-value		5.0 × 10^−7^ **	ND	ND	ND	ND	ND
% < median	33	75					
% fused	0	36					
Leaf 4	5.1 ± 0.6 (48)	3.7 ± 1.5 (56)	5.1 ± 0.4 (24)	5.0 ± 0.2 (26)	4.5 ± 0.9 (24)	10.0 ± 1.2 (5)	5.0 ± 1.9 (5)
*p*-value		3.1 × 10^−9^ **		9.7 × 10^−2^	2.6 × 10^−3^ **		8.2 × 10^−4^ **
% < median	6	54	0	4	29	20	100
% fused	0	32	0	0	8	0	0
Leaf 5	5.1 ± 0.5 (48)	4.6 ± 0.9 (47)	5.3 ± 0.5 (24)	5.0 ± 0.2 (26)	4.1 ± 1.2 (24)	15.4 ± 1.9 (5)	5.0 ± 2.5 (5)
*p*-value		1.0 × 10^−3^ **		3.8 × 10^−2^ *	1.0 × 10^−4^ *		4.8 × 10^−5^ **
% < median	0	23	0	0	46	20	100
% fused	0	4	0	12	8	0	20
Leaf 6			5.3 ± 0.7 (23)	4.8 ± 1.2 (26)	4.0 ± 1.4 (22)	15.2 ± 1.6 (5)	6.4 ± 3.5 (5)
*p*-value	ND	ND		4.7 × 10^−2^ *	2.9 × 10^−4^ **		1.3 × 10^−3^ **
% < median			4	12	45	40	100
% fused			0	27	23	0	20
Leaf 7			5.6 ± 0.8 (24)	4.4 ± 1.9 (25)	3.3 ± 2.3 (15)	16.0 ± 0 (5)	10.4 ± 5.1 (5)
*p*-value	ND	ND		4.2 × 10^−3^ **	8.9 × 10^−4^ **		3.5 × 10^−2^ *
% < median			0	32	67	0	80
% fused			0	56	73	0	60

## Data Availability

Data are contained within the article and Supplementary Materials.

## Supplementary Materials

The following supporting information can be downloaded at: https://www.mdpi.com/article/10.3390/plants13182566/s1, Figure S1: Computer simulations of the effect of PAT inhibitor treatment on leaf margin complexity; Figure S2: Exogenous IAA affected leaflet formation in tomato (variety Glamour); Text S1: Computational Methods. References [44,45,46] are cited in the supplementary materials
