# Supplementary material for: The Effects of Auxin Transport Inhibition on the Formation of Various Leaf and Vein Patterns
Source: Plants (Basel). 2024 Sep 12;13(18):2566. doi: 10.3390/plants13182566 (PMC11434698; doi:10.3390/plants13182566)
Supplement: Supplementary file 1 [file plants-13-02566-s001.zip › Supplementary Text S1.pdf]

Supplementary Text S1, Computational Methods

VirtualLeaf downloading, installation and usage is described in [42, 44–46]. VirtualLeaf v1.0.2 was used in the current work. Polar auxin transport (PAT) was modelled as in Holloway and Wenzel ([35], Table 1 parameters). Leaf growth was simulated by cell expansion and division in the margin cells. Expansion had two contributions: a) cell areas expanded by a fixed amount per growth step according to position along the margin – 0 in the terminal lobe, 0.6 in the lateral lobes, and 0.5 in the base of the leaf (initial cell areas are between 250 and 300 computational units); and b) cell areas expanded depending on their auxin concentration [A], with a Hill relation $\frac{[\mathrm{A}]^6}{5+[\mathrm{A}]^6}$, where the dissociation constant (5) corresponds to half-maximal growth at [A] = 1.3. Auxin was synthesized in margin cells from a precursor, A_{prec}. The earlier development of the terminal lobe was modelled by $A_{prec} = 0.5$ at $t = 0$, with $A_{prec} = 0$ in the lateral lobes until 2h47m (computational units). A_{prec} increased in all margin cells by 1×10^{-4}/s to a maximum of 1.3. Cells divided when they doubled in area and cell shapes minimized overall elastic energy.

Code is available at https://github.com/davidhollowaybc/complexleaves, with the initial leaf specification in complexleaf_0h.xml and the VirtualLeaf code module in complexleaf.cpp and complexleaf.h.

\
